# Children and adolescents‘ views on artificial intelligence in pediatric healthcare: a qualitative focus group study

**DOI:** 10.1186/s12887-026-07121-w

**Published:** 2026-06-13

**Authors:** Lisa Reinhart, Janna-Lina Kerth, Anne C. Bischops, Maurus Hagemeister, Lisa Krassuski, Ertan Mayatepek, Thomas Meissner

**Affiliations:** 1 Department of General Pediatrics, Neonatology and Pediatric Cardiology, Medical Faculty, University Hospital Duesseldorf, Heinrich-Heine-University, Moorenstr. 5, Duesseldorf, 40227 Germany; 2 Computational Health Informatics Program, Boston Children’s Hospital, Boston, MA USA

**Keywords:** Artificial intelligence, Pediatrics, Healthcare, Children and adolescents, Qualitative research, Focus group interviews

## Abstract

**Background:**

The increased use of artificial intelligence (AI) in healthcare, including pediatrics, offers significant opportunities for improving quality, personalization, and effectiveness of care. Nevertheless, successful implementation of this approach in pediatric patients depends on their acceptance and the integration of their preferences regarding AI use within participatory decision-making. Since previous research insufficiently addresses the perspectives of children and adolescents on AI use in pediatric healthcare, the objective of this study was to address this research gap.

**Methods:**

This qualitative study was conducted at the University Children’s Hospital Duesseldorf, Germany, and in surrounding schools. Recruitment took place from March to September 2024. Semi-structured interviews including two scenarios were conducted: an AI-based application (1) for assessing the development of children and adolescents, (2) for supporting and monitoring children and adolescents with chronic conditions. Content analysis was used to evaluate information.

**Results:**

Eighteen focus group interviews with 58 participants (8–11 years = 10; 12–14 years = 23; 15–17 years = 25) were conducted. Nine key topics emerged: benefits, fears, dealing with misdiagnoses of AI, responsibility for data input and access, impact on the physician-patient relationship, the right not to know, transparency, data sources, and desired features. Participants repeatedly emphasized that AI should serve only as a supportive tool, with healthcare professionals retaining final authority and critically evaluating its findings. Key benefits included early detection of health anomalies and reduced diagnostic or human errors. Barriers to acceptance encompassed data privacy concerns, suspected lack of empathy, absence of physical examination, and risk of false diagnoses.

**Conclusions:**

This qualitative study underlines, that the requirements, perceived benefits, and concerns of AI-based applications in pediatric healthcare are multifaceted and necessitate including minors in their development and implementation. Age-appropriate education on AI use, risks, limitations, opportunities and strengths is essential for acceptance. If possible, minors should be openly informed about the limitations of AI decision transparency, with underlying mechanisms explained where appropriate. At best, children and adolescents should already be involved in development of AI applications in terms of information and consent.

**Trial registration:**

This study was retrospectively registered in the German Clinical Trials Registry (DRKS00034474; 17th June 2024).

**Supplementary Information:**

The online version contains supplementary material available at 10.1186/s12887-026-07121-w.

## Background

Artificial intelligence (AI) is increasingly being used in healthcare. With the ongoing digitalization, AI holds significant potential to enhance the quality, efficacy, and personalization of medical care [1–6]. AI technologies could assist healthcare professionals or patients, provide more accurate diagnoses and create personalized treatment options [2, 4, 5, 7].

Potential applications for AI also exist in pediatrics, for example in monitoring child development, diagnostic processes, and the treatment of acute or chronic diseases [2, 8–10]. Despite this potential, the successful use of AI in pediatrics highly depends on the acceptance of the stakeholders and their consent to its use. Research on the acceptance, its short- and long-term effects and effects of restricted autonomy in pediatric care is scarce, especially regarding the views of children and adolescents [9–11]. In the very few studies yet available AI holds potential to effectively support home-based therapy, despite concerns regarding reduced human interaction and potential technical limitations, and its acceptance depends largely on its effectiveness and supervision by healthcare professionals [2, 12, 13]. So far, children and adolescents would like their preferences to be considered regardless of their capacity to consent and limited patient autonomy [2, 12, 13].

The long-term implementation of AI in pediatrics requires a comprehensive understanding of the perspectives of minors as they form the affected target group. Since children and adolescents differ significantly from adults in terms of needs, stage of development and understanding of medical processes, their perspectives, concerns, and expectations should be actively considered in terms of participation when developing and implementing AI-based applications in pediatrics.

The objective of this study is therefore to provide a conceptual basis for further implementation of AI in pediatrics by gaining insights into the perceived opportunities, challenges, expectations, and concerns by children and adolescents associated with its use.

## Methods

### Study design

This qualitative, monocentric study was conducted at the University Children’s Hospital Duesseldorf using focus group interviews as a suitable method to explore participants‘ perspectives. The study data is reported in accordance with the Consolidated Criteria for Reporting Qualitative Research (COREQ) checklist [14].

This study was part of the interdisciplinary research project „AI-PHCA – AI-based Preventive Healthcare for Children and Adolescents“.

### Research team

The research team consisted of four female and two male pediatricians and two student assistants working at the University Children’s Hospital Duesseldorf.

### Recruitment, sample and setting

Children and adolescents were recruited between March and September 2024 at the University Hospital Duesseldorf, a comprehensive school in Duisburg, and a vocational college in Cologne, or through private contacts. Participants received a financial compensation (10–20€, age dependent). Focus group discussions were conducted in person in a conference room in the hospital or in classrooms.

Inclusion criteria for study participation were age 8–17 years, sufficient German language proficiency, adequate general health for participation (e.g., no need for monitoring or quarantine when recruited at the hospital), and a declaration of consent from the study participants and their parents. Exclusion criteria included no consent and insufficient language skills to engage in focus group interviews.

Participants were informed that participation in the study was voluntary without advantages or disadvantages. Withdrawal from the study was possible at any time without consequences.

### Data collection

Data was collected in focus group interviews. The interviews were conducted using a semi-structured interview guideline including two scenarios with open-ended questions: an AI-based application (1) for assessing the development of children and adolescents (2), for supporting and monitoring children and adolescents with chronic conditions (see supplementary material). The research team developed a specific interview guideline for each age group, adapted in level of complexity and language style (see supplementary material). Each interview guideline was pretested and reviewed afterwards.

The interviews were moderated by physician members of the research team (LR, AB, LK, MH) while a second person (physician or student assistant) was taking notes. Interviews were recorded by an audio device after obtaining participants‘ and parents’ consent. At the beginning, moderators and participants introduced themselves, followed by a brief study overview and group rules such as valuing different opinions, avoiding overlapping speech, and silencing phones. Participants might have had prior contact to moderators if they had been recruited from their personal networks or were patients at the hospital. Participants were recruited until arguments reached saturation. No repeat interviews were conducted. Participants did not receive access to interview recordings or notes.

### Data analysis

The focus group interviews were audio-recorded, pseudonymized and transcribed professionally. Transcripts were analyzed using MAXQDA (MAXQDA, Software for qualitative data analysis, 1989–2025, VERBI Software. Consult. Sozialforschung GmbH, Berlin, Germany) and evaluated using Mayring’s inductive coding method [15]. Each transcript was coded independently by two physician members of the research team (LR, MH, JK). At least one coder was not involved in the respective interview. Coding systems for each age group were developed after reviewing several transcripts (defined as at least two interviews of each group) and applied to the remaining ones (LR, JK, MH). Any discrepancies in coding were discussed, clarified, and adjusted accordingly (LR, JK, MH). The resulting codes were assigned to corresponding categories to develop a coding framework. Quotes were translated using DeepL Translator (DeepL SE, Cologne, https://www.deepl.com/de/translator).

## Results

This study comprised a qualitative analysis of focus group interviews with children and adolescents in which perspectives toward AI in pediatrics were examined.

A total of 17 focus group interviews were conducted between April and September 2024 (see Table 1 for characteristics). They took place at the University Children’s Hospital Duesseldorf or at schools in Duisburg and Cologne and lasted 30–90 min. Recruitment of study participants was terminated once arguments reached saturation. Some participants were already acquainted when they attended the same school. In one interview with children, two parents were present.

**Table 1:** Characteristics of focus group interviews

Group	Age (in years)	Number of participants	Number of focus group interviews	Number of participants in each focus group interview
Children	8–11	10 (male = 1, female = 9)	4	1; 2; 2; 5
Younger adolescents	12–14	23 (male = 11, female = 12)	6	1; 3; 3; 4; 6; 6
Older adolescents	15–17	25 (male = 13, female = 12)	8	1; 1; 2; 2; 4; 4; 5; 6

Most participants, especially adolescents, had experience with digital technologies (e.g., mobile phone use), health-related applications (e.g., tracking of calories) and some were already familiar with AI (e.g., through its application in homework research). The participants’ socioeconomic background was not explicitly recorded, but recruitment from diverse settings (hospital, schools, private contacts) enabled a highly probable variation. Pre-existing health conditions were not recorded unless voluntarily reported by study participants.

**Fig. 1:**
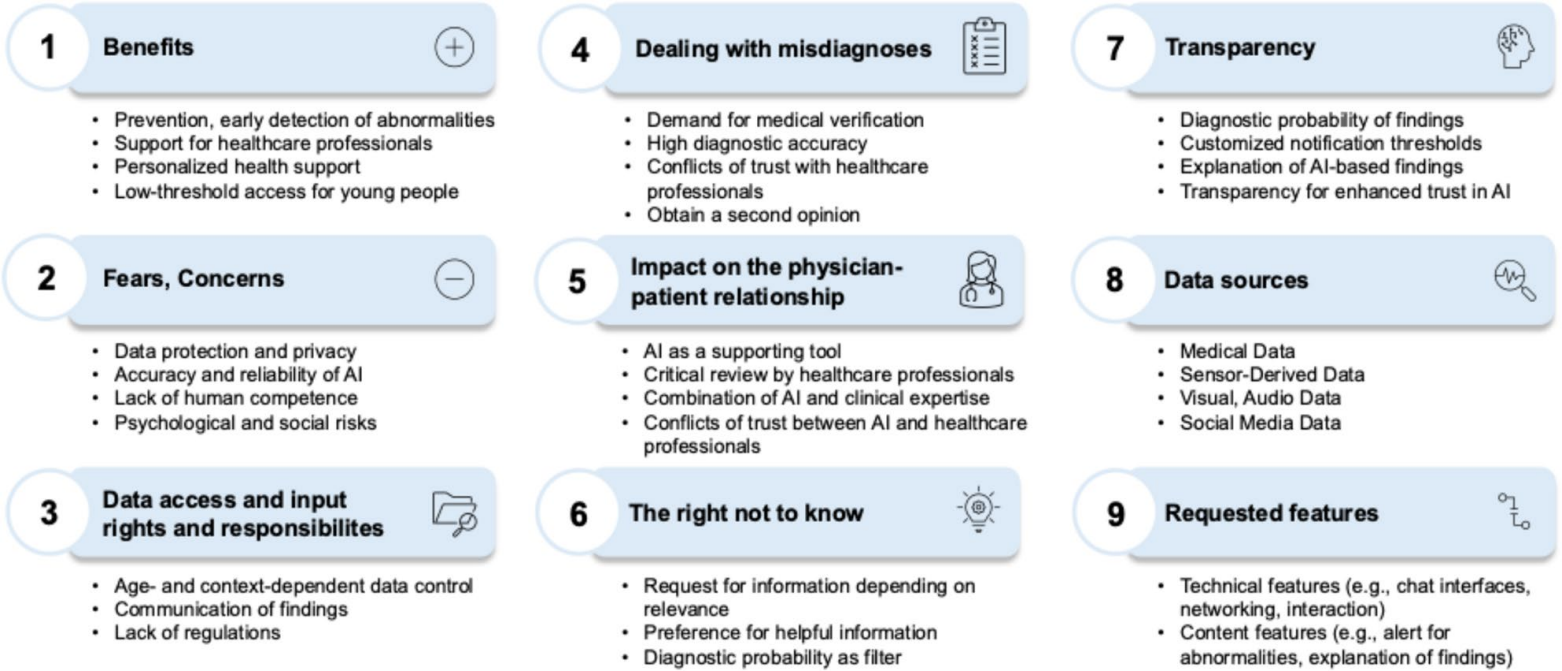
Overview of categories and key findings

**Fig. 2:**
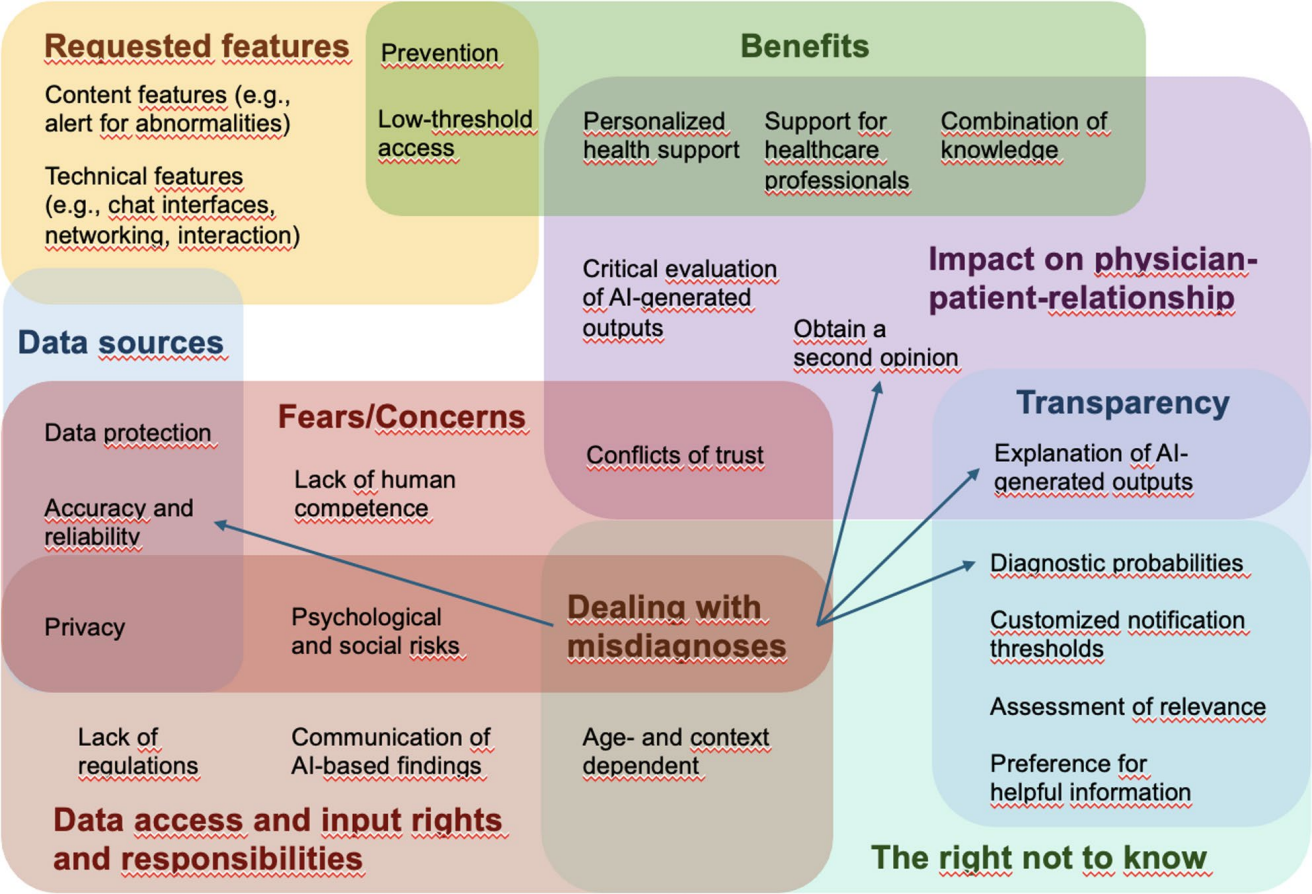
Overview of code connections and intersections

The interactive setting encouraged discussion and revealed diverse perspectives on the topic. When evaluating the interviews nine content categories emerged (see Fig. 1). Figure 2 shows the connections of recurring themes and areas of overlap. Table 2 shows participant quotes by category.

**Table 2:** Quotes from study participants in the respective age groups on different categories

#	Category	Group	Quotation
1	Benefits	Children	“Well, I think it’s pretty important (…). If an app like that told me I needed to go to the doctor, then I would just do it. Because it’s better to go to the doctor once too much and then go to the doctor unnecessarily than to go to the doctor once too little and then something happens.”
2		Younger adolescents	“Maybe you just overlook something. I think software like that would simply go through all the possibilities and couldn’t do it any more accurately than a human being can at present.”
3	Fears and Concerns	Older adolescents	“And I would also be really afraid that it wouldn’t just be listening in on my sleep or something like that, but really everything, like phone calls, conversations I have with my friends or whatever, because you don’t know. And it could be that a cell phone is turned off, but the app is still running. Yes, I would be really scared of that. And it’s possible that the data is being passed on somehow. Yes, and I don’t know what happens to my data, my voice, these recordings, or the information I enter into the app. So it’s actually like social media, where there are also pictures, and I don’t really know what happens to them because they’re on the internet. But I think that’s different, because with social media, I know what I’m posting, I decide that. With the app, I don’t know.“
4		Younger adolescents	“I think one limit, for example, would be accessing your own gallery, because sometimes it doesn’t just contain pictures of yourself, but also selfies with friends or pictures that friends have sent you, and that could lead to a false result, because the AI might pull in something with another person in it. And secondly, if I agree that things belonging to ME can be used, that doesn’t mean that things belonging to other people can be used. So that would definitely be the limit for me.“
5		Younger adolescents	“Let’s say (…) you’re walking (…) through the city center and someone next to you is smoking (…). The app then detects that you have inhaled nicotine or something else, and this information is sent to your parents. Your parents confront you, asking if you’ve been smoking or something, even though you were just walking through the city center.”
6		Older adolescents	“Yes, because that’s just not human. Those aren’t feelings, and that wouldn’t reassure me. I would still feel that something is wrong and that I need to see a doctor.”
7	Data Access and Input Rights and Responsibilities	Younger adolescents	“That’s why I think that, at least from a certain age, you should be able to decide for yourself. (…) And maybe you can’t use it before that age, or your parents have to decide. But actually, I don’t think it’s up to parents to decide.”
8		Children	“Because it’s no use if the child knows about it and says, “I want to keep it to myself for now.” Then the illness can get worse and your parents can’t do anything about it because they don’t know.”
9		Older adolescents	“It doesn’t really matter who tells you that, but rather who helps you afterwards, who reassures you afterwards, or who accompanies you through it.”
10	Dealing With Misdiagnoses	Older adolescents	“Sure, I would want to know, but if I got the information from the app, I would take it to the doctor at the hospital and have them clarify whether it’s really true or not.”
11	Impact on the Physician-Patient Relationship	Younger adolescents	“I would also say that AI does not make the really important decisions at this point, but rather that doctors use AI to support them, and then make the decisions about what is really important next (…) themselves. I think it’s also a question of responsibility if something goes wrong. If AI does that, then you never know who is responsible if something goes wrong.”
12		Younger adolescents	“You tend to have a more personal relationship with your pediatrician because they are a human being. It’s still different from AI. It would be a different relationship, but I would also be open to AI. However, I would interact with AI differently than I would with my pediatrician.”
13		Younger adolescents	“I wouldn’t have as much confidence in the doctor anymore. (…) I wouldn’t feel as comfortable if I knew that the doctor was only getting information from AI and no longer relying on his own knowledge or that of other doctors.”
14		Children	“A doctor, because an app can sometimes malfunction. Sometimes it gets things mixed up or… so I would rather believe the doctor. They can examine you thoroughly, and the app doesn’t have the same capabilities as a doctor or something like that. I would rather believe the doctor.”
15	The Right Not To Know	Older adolescents	“Then you start worrying about something, which I really wouldn’t wish on anyone, but it could be that you die early in a car accident or something else. And then you’ve been worrying the whole time and haven’t even gotten to the point of whether it’s true at all.”
16		Older adolescents	“Or you think the whole time that you’re going to get this disease, and in the end you don’t get it at all because somehow it was wrong.”
17	Transparency	Younger adolescents	“I also think that you actually… well, of course, above 50% it’s more likely, but it can also be as low as 10% or so that you always get a message, but then maybe there are different warning levels, so below 50% it’s not that likely, or (…) that you get an appointment with the doctor right away.”
18	Potential Data Sources	Younger adolescents	“So, I think with social media (…), I don’t think it would be a good idea for AI to have access to social media accounts, because some people might be a bit pretentious on social media and don’t always tell the truth, and that could perhaps distort the course of the day a little bit (…), because not everyone always shows their true self on social media.”
19		Older adolescents	“Because I don’t think (social media) is important for AI, and as was just said, it’s simply data that others could potentially exploit and also sell. And for me, that has nothing to do with health, what videos I watch. Maybe how much time I spend watching videos, but not specifically what I watch.”

### Benefits

Early warnings and alerts for abnormal findings were frequently cited as beneficial to AI-based applications among all age groups (Table 2; quote #1). Older adolescents believed AI could contribute to early disease detection and prevention with short- and long-term benefits. According to adolescents AI-based applications were considered helpful for acute and chronic diseases by assisting clinicians, facilitating or reducing medical visits, and improving quality of life. Furthermore, participants of all age groups noted AI’s role in disease management, guidance, adherence, and real-time feedback (e.g., glucose monitoring) as well as combining AI and clinician expertise.

According to younger adolescents AI could reduce the burden on healthcare professionals by giving preliminary advice, performing routine tasks, reducing errors or functioning as a screening tool (Table 2; quote #2). Older adolescents highlighted AI’s potential in sensitive areas such as mental health. AI’s anonymity, its associated low threshold for asking questions, continuous availability, and the lack of being judged were considered advantageous and promoted greater openness toward AI across all ages.

### Fears and concerns

Across all interviews, major fears were data misuse, lack of data security, loss of privacy or risks of sharing sensitive information if data is “*being passed on somehow*” (Table 2; quote #3). Among adolescents concerns extended to uncontrolled data recording and affecting third parties (e.g. exposing friends‘ images; Table 2; quote #4).

All age groups questioned AI’s diagnostic accuracy and reliability, citing risks of false positive or negative diagnoses, irrelevant alerts, and delays in seeking medical care. According to younger adolescents data input errors or potential confounders in image evaluation (e.g. camera quality) “*could lead to a false result*” (Table 2; quote #5).

Furthermore, adolescents in general feared reliance of healthcare professionals on AI without using their clinical experience and highlighted AI‘s lack of empathy and clinical intuition. They stated AI “*doesn’t have the same capabilities as a doctor*” because many conditions cannot be diagnosed via images or without physical examination (Table 2; quote #6 and #14).

Older adolescents also stated psychological risks, including increased anxiety, stress from continuous monitoring, and app dependency. Younger adolescents feared limited support in using AI regarding responsibility for care and decision-making. Adolescents feared social isolation when receiving AI-based abnormalities, raising legal and ethical concerns, and fears of replacing human care, misuse, or technical failure.

### Dealing with misdiagnoses

All age groups emphasized that AI-based diagnoses require verification by healthcare professionals to “*clarify whether it’s really true or not*” (Table 2; quote #10). Automatic forwarding to doctors could accelerate care. High diagnostic accuracy of AI-based applications was desired among all ages to avoid anxiety with acceptable certainty levels varying by disease severity, e.g. lower for mild and higher for serious diseases according to adolescents. Expressions were more detailed with increasing age. Adolescents feared AI might challenge trust in healthcare professionals by raising concerns that they might miss something that AI identified.

### Data access and input rights and responsibilities

Views regarding responsibility for data input and access varied from exclusive user access to broader stakeholder involvement and led to controversial discussion in all age groups. Parental access was considered appropriate for minors given limited autonomy and understanding, yet independence was emphasized by children and especially adolescents (Table 2; quote #7). Participants of all age groups agreed that critical findings should automatically involve stakeholders to provide support. The transition from parental to independent use represented a key challenge. Decisions regarding data input and access were considered age- and context-dependent and revealed differing opinions across age groups. Participants saw parental oversight as a safeguard for data input while concerns among adolescents included excessive parental data sharing, negative consequences, and trust issues concluding that data sharing should be controlled by users (Table 2; quote #8).

Notification preferences varied by age and diagnosis with a general preference for early alerts. Most participants preferred clinicians communicating AI findings to parents prior to children citing greater empathy and reassurance (Table 2; quote #9). Older adolescents favored access to interpretations of AI and healthcare professionals.

### Impact on the physician-patient relationship

Younger adolescents viewed AI as a complementary tool to clinical expertise by reducing workload and increasing efficiency without fearing loss of medical competence (Table 2; quote #11). Participants requested clinicians to critically evaluate AI-based results and wishing “*doctors to use AI to support them*,* and then make the decisions (…) themselves*” (Table 2; quote #11). Concerns among adolescents included fears of AI inferiority, depersonalization, and reduced interaction (Table 2; quote #12). AI use in consultations could infer reduced clinical competence and lower perceived consultation value (Table 2; quote #13). Trust in AI would increase if healthcare professionals repeatedly confirmed its results.

Discrepancies between AI and clinician statements were seen as critical to the physician-patient relationship across all age groups. Younger adolescents generally trusted physicians more, whereas some older adolescents expressed greater trust in AI when uncertainties persisted (Table 2; quote #14). To address this discrepancy, participants preferred consulting a third opinion or additional medical testing.

### The right not to know

When asked whether to receive all AI-based information, including potential future or untreatable conditions, adolescents emphasized assessing clinical relevance, distinguishing minor diseases from serious diseases, e.g. a future cold differed from a potential dementia diagnosis. Adolescents rejected information with little practical value to avoid distress and preferred notifications only above a certain probability threshold, valuing information for prevention and health awareness (Table 2; quote #15, #16).

### Transparency

All age groups agreed that AI-based findings should include probability estimates with notification thresholds varying by perceived disease severity (Table 2; quote #17). For critical diseases, AI should provide notifications at low probabilities.

Many participants, especially older adolescents, requested explanations of AI conclusions, including potential errors, data sources, and algorithmic foundations. Others preferred optional, simplified explanations. Younger adolescents valued transparency about data access and use to build trust and self-assessment.

### Potential data sources

A list of potential data sources named in the interviews is provided in Table 3 (Table 2; quote #18, #19).

**Table 3:** List of potential data sources named in the focus group interviews

Potential Data Source	Examples named in the interviews
Medical Data	• Age • Preexisting illnesses • Family history • Laboratory results • Medication, substance use (e.g., intake frequency, doses) • Development parameters (e.g., height, weight) • Mental health information
Sensor-Derived Data	• Physical activity (e.g., step counts, movement, leisure activity) • Sleep data • Vital parameters (e.g., heart rate, temperature) • Blood glucose
Visual and Audio Data	• Photos (e.g., photos of faces or body to detect skin abnormalities, photos of food to analyze ingredients or calories) • Video recordings (e.g., to analyze movement) • Voice recordings (e.g., to assess speech development)
Social Media and Messaging App Data	• Uploaded content (e.g., photos, videos, texts) • Consumed content • Written content (e.g., comments, text messages) • Screen time
Manual Input	• Nutrition details • Diary (e.g., to assess pain or sleep) • Symptom questionnaire
Social Data	• School performance

### Requested features

Participants identified preferred features for AI-based applications that are listed in Table 4.

**Table 4:** List of requested features of AI-based applications named in the focus group interviews

Category	Requested Features
Technical Features	• Chat interfaces (e.g., with AI or healthcare professionals) • Expert networking • Appointment scheduling • Voice interaction • User-friendly navigation • Manual data input • Consent for data use and sharing • Indicators for active data collection • Predictive modeling • Connecting profiles (e.g., with family, friends, other persons affected) • Language selection • Emergency call feature
Content Features	• Alert for abnormalities, automatic alerting of people (e.g., caregivers, healthcare professionals, friends) • Explanation of abnormal findings • Provision of probability estimates • Recommendation of consultations at diagnostic thresholds • Reminder feature • Symptom tracking • Compilation of health data for healthcare professionals

## Discussion

This study identified multiple perspectives of how minors perceive AI in pediatric healthcare. Key findings revealed diverse perceived benefits, including early detection of health anomalies, improved care for acute and chronic conditions, and knowledge expansion through the combination of clinical expertise and AI. However, fears regarding data protection and incorrect findings were also present. Therefore, participants demanded healthcare professional verification of AI-generated findings, and requested high safety standards regarding diagnostic accuracy, clear regulations on responsibilities and liability for AI for future application.

Although the views of younger and older adolescents were relatively similar, developmental differences emerged between age groups on the discussed topics, suggesting an increasing understanding of AI and healthcare with age, as well as differences in cognitive abilities regarding long-term implications and adoption of AI.

Participants across all age groups identified key advantages of AI in detecting overlooked abnormalities and improving data analysis and diagnoses [16]. They saw AI as facilitating personalized medicine through individualized treatment adjustments and enabling timely detections of changes through automated alerts [4, 16]. Consistent with previous studies, our participants generally accepted AI as an assistive tool in clinical decision-making that reduces errors, improves medical care, and shortens waiting times, but rejected its exclusive use [6, 12, 16, 17]. Participants hoped AI would give healthcare professionals more time for their patients, noting it should not replace them [6, 16, 17].

Acceptance of AI varied with perceived disease severity, especially among adolescents [6, 16]. Unlike prior studies reporting parental openness for minor conditions, children and adolescents highlighted AI’s beneficial use for serious and chronic illnesses [16].

Similar to prior studies our participants considered AI-based applications for home monitoring of chronic diseases (e.g., diabetes) beneficial to increase autonomy, improve quality of life, and reduce time expenditure [4].

In all interviews, privacy protection and confidentiality of sensitive health data represented central concerns, aligning with previous studies [8, 17, 18]. Concerns about potential commercial data use and lacking regulations for addressing discrepancies represented potential barriers to its acceptance across all age groups underscoring the need to consider children’s and adolescents‘ preferences in AI development and implementation [12]. Similar concerns were expressed by parents, suggesting possible influence on participants’ views [4, 6, 8, 17]. Addressing these issues requires clear guidelines, transparency regarding AI use, and source references “*if something goes wrong*” (quote #11) [4]. Age-appropriate education and information is essential to enable informed decision-making about AI, especially among younger age groups.

Additional concerns among adolescents included AI’s inability to perform physical examinations and limited knowledge of long-term consequences [19]. Participants repeatedly expressed ethical concerns about diagnostic errors or incorrect treatment recommendations, raising awareness for adequate user training [16, 20]. An increased diagnostic accuracy by AI compared to current methods, proven therapeutic efficacy, physician trust, human oversight and clinical validation may enhance AI acceptance [2, 3, 6, 12, 21]. Individual cross-thematic response patterns indicated that greater anxiety toward AI was associated with increased trust in physicians; while more fears and concerns were expressed, this did not necessarily lead to a stronger demand for transparency.

The influence of AI on the physician-patient relationship appeared to depend on the extent of its use. Participants debated whether to trust AI or clinicians when opinions differed, but agreed that clinicians should retain ultimate control, reflecting underlying mistrust in AI. Consistent with prior studies, participants stressed that medical decisions should not rely exclusively on AI assessments but involve clinical evaluation [1, 6, 16, 18]. Like findings from previous research involving parents, all age groups valued shared decision-making, transparency about AI use and prior consent [6, 8, 12, 13, 16, 18]. While most adolescents preferred human-led decisions supported by AI and feared reduced clinician-patient interaction, some showed greater trust in AI outputs than reported previously indicating a shift in AI perception [7, 13, 18]. Others questioned AI-based recommendations, reflecting variability in user competence and risk of misinterpretation. Our results reinforce prior findings emphasizing the need for regulations and safety measures in AI use [12, 17].

Most participants across all age groups rejected exclusive parental use of AI-based applications, favoring active involvement primarily due to privacy concerns. They emphasized individual preferences should be respected regardless of age and wished to participate in developmental processes as also stated in prior studies [13, 17]. An appropriate age for minors to independently consent to AI use in healthcare remains unclear, while each age group considered their own age as appropriate, underscoring the need for regulations, including cases of later withdrawal [4, 11]. Adolescents distinguished “relevant” from “irrelevant” information when discussing automatic data sharing, expecting AI to assess clinical relevance and reflecting a more advanced comprehension of AI-generated data compared with children [21].

Insufficient support when communicating abnormalities was viewed as potentially anxiety-inducing, stressing the importance of adequate training to ensure appropriate interpretation and use, and clarifying whether abnormalities should be communicated by AI, caregivers, or healthcare professionals [4]. Adolescents considered mental health issues particularly sensitive and raised doubts about AI’s empathy and ability to deliver bad news appropriately, suggesting more advanced cognitive abilities with increasing age, as well as a better understanding of ethical considerations in clinical communication and interpersonal aspects of care [17].

Most participants preferred to be informed about AI-derived findings only if they were treatable or preventable. Among all age groups acceptance was high in terms of prevention while older age groups articulated more detailed considerations regarding its implementation.

Participants‘ desire for transparency varied depending on their prior knowledge of AI and slightly increased with age. Greater understanding of AI-systems may enhance user acceptance [8, 18]. Our participants wanted to know whether AI is used in their care without explicitly demanding detailed explanations contrasting prior reports and highlighting the importance of transparency and participatory decision-making [6]. Although participants did not explicitly mention that healthcare professionals should fully understand AI, they considered their evaluation of its findings important.

The discussions about potential data sources were controversial and revealed varying degrees of openness toward different AI applications across all ages aligning with prior studies [17]. While the collection and analysis of sensor-derived data (e.g., movement, sleep data) and nutrition was generally well accepted, images and videos were viewed critically due to privacy and data quality concerns. Openness to AI and data sharing was influenced by perceived user benefit and e.g., image type (e.g., food vs. people). Adolescents raised uncertainty whether AI-systems could compensate for user-generated variability in data quality, indicating a more advanced understanding of AI-systems compared with children.

Despite existing approaches in evaluating social media data, adolescents largely rejected their use due to privacy concerns [22, 23]. The interviews revealed a discrepancy in data disclosure: sharing data on social media was viewed voluntary and self-determined, unlike in AI-based applications. Additionally, adolescents noted that social media content is rarely truthful, questioning health relevance (quote #3). When informed about promising approaches to detect depression and suicidal tendencies, participants generally became more receptive, highlighting disease-specific differences in acceptance and underscoring the need to involve young people in developing AI-based tools.

Ease of use, adapting to user preferences (e.g., setting probability thresholds for AI-based notifications) and simplified explanations of AI-based outputs could increase acceptance across all age groups [24]. Explicit consent and clear data collection indicators were deemed essential.

A key strength of this study is the inclusion of children and adolescents, an understudied group regarding AI acceptance in healthcare. Including participants from different age groups allowed for a broad representation of attitudes and concerns, while developmental differences could have influenced AI-perceptions. Recruiting participants from clinical and non-clinical settings enabled a comprehensive assessment of AI use. The broad range of discussed topics provided an extensive understanding of participants‘ perspectives. Most focus groups were conducted without parents to minimize response bias. The presence of parents may have influenced responses in one interview with children.

Group dynamics and composition, preexisting relationships, social desirability, and initial arguments may have affected discussions and limited participation. Non-random sampling likely favored motivated participants, and the gender imbalance among children, as well as the limited number of participating children, may have influenced results and limited the validity and generalizability of interpretations. Recruiting children proved difficult because there were few children within the same age group who were present and willing to participate in the study, and because they also had a shorter attention span and limited knowledge about AI. The limited number of participants in some focus groups restricts the diversity of the discussion and the generalizability of the findings. Pre-existing conditions among study participants (e.g., when recruited in the hospital) may have influenced their attitudes toward AI in healthcare. Moderator behavior (e.g., tone, body language) and clinical constraints (e.g., isolation, monitoring) may have impacted recruitment and responses. The moderation of the focus group interviews by physicians may have influenced the participant’s responses.

## Conclusion

The results show that AI was accepted as a supportive tool, though participants demanded its outputs to be reviewed by healthcare professionals. Great potential is seen in prevention, enhanced monitoring, increased knowledge by combining AI and medical expertise and the improvement of care. Major barriers to its acceptance are concerns regarding data privacy and incorrect diagnoses. Data protection, transparency, and regulations for minors who cannot give informed consent, as well as age-specific information provision and guidance must be considered when implementing AI in pediatric care. This study highlights that the acceptance of AI by children and adolescents and their willingness to share data and engage with AI-systems is essential for its successful implementation in pediatric healthcare.

## Supplementary Information

Supplementary Material 1.

Supplementary Material 2.

Supplementary Material 3.

Supplementary Material 4.

## Data Availability

The results of the focus group participants‘ responses cannot be disclosed due to data protection and confidentiality requirements, except as reported in this paper. The guidelines for the semi-structured interviews are available in the appendix.

## Abbreviation

AI: Artificial intelligence
