# Supplementary material for: Children and adolescents‘ views on artificial intelligence in pediatric healthcare: a qualitative focus group study
Source: BMC Pediatr. 2026 Jun 13;26:563. doi: 10.1186/s12887-026-07121-w (PMC13267202; doi:10.1186/s12887-026-07121-w)
Supplement: Supplementary file 1 — Supplementary Material 1. [file 12887_2026_7121_MOESM1_ESM.pdf]

BMC Pediatrics

Children and adolescents‘ views on artificial intelligence in pediatric healthcare: a qualitative focus group study

Lisa Reinhart, MD[1]; Janna-Lina Kerth, MD[1]; Anne C. Bischops, MD[1,2]; Maurus Hagemeister, MD[1]; Lisa Krassuski, BA, MD[1]; Ertan Mayatepek, MD[1]; Thomas Meissner, MD[1]

Affiliations:

1 Department of General Pediatrics, Neonatology and Pediatric Cardiology, Medical Faculty, University Hospital Duesseldorf, Heinrich-Heine-University, Duesseldorf, Germany

2 Computational Health Informatics Program, Boston Children’s Hospital, Boston, MA, USA

Address Correspondence to:

Lisa Reinhart, Department of General Pediatrics, Neonatology and Pediatric Cardiology, Medical Faculty and University Children’s Hospital Duesseldorf

Moorenstr. 5, 40227 Duesseldorf, Germany

Email address: lisa.reinhart@med.uni-duesseldorf.de

Phone: +49 211 81-00

Supplementary Material: Semi-structured interview guideline with children

Focus group discussion

„Acceptance of AI applications in healthcare – children“

A) Introduction

Thank you very much for coming today. My name is XXY, I work at the University Children’s Hospital Duesseldorf, and I will be asking a few questions today. This is XXY. She/he will be taking notes during the conversation.

We are working on a project that, among other things, is researching how new technologies can help pediatricians provide better care for children and adolescents. These new technologies primarily involve what is known as artificial intelligence.

We want to know what different people think about artificial intelligence. For example, what they like about it and what they dislike about it. To find out, we talk to pediatricians, pediatric nurses, parents, children, and teenagers.

Great to have you here today!

Now I'll explain a few things about how the discussion will proceed.

The discussion will last about half an hour to an hour.

Participation in the discussion is voluntary. You can stop the conversation at any time.

The discussion is not an exam or a test. There are no right or wrong answers. Just say what you think and feel freely!

It is important that only one person speaks at a time.

I have prepared some questions for you. If you think of any other important issues, you can raise them at any time.

Everything that is said during the discussion will be treated confidentially by our team. This means that no one except the people in our team will find out what you say. We ask that you also refrain from telling anyone else what was said during the discussion.

At the beginning of the discussion, everyone should introduce themselves by their first name. However, we will anonymize all data later. This means that no one will be able to find out who participated in the discussion.

XXY will take notes during the discussion. We will also record the conversation with an audio device. Are you all okay with that? I will turn on the device now.

Do you have any questions before we begin the discussion?

Two more notes: Please turn off your cell phones during the discussion or set them to silent mode. And please help yourselves to drinks and cookies if you would like some.

B) Opening

Let's start by briefly introducing ourselves.

My name is XXY (discussion leader), I am XXX years old and I work as XXX. My name is XXY (co-moderator), I am XXX years old and I work as XXX.

1. What are your first names? How old are you?
2. Do you have a cell phone? Do you know what an app is?
   a. If necessary, briefly explain the term: An app is a computer program that is mainly used on cell phones or tablets. There are many different apps that can be used to do a wide variety of things. For example, there are apps for playing games, music apps, apps for chatting, and apps that tell you what the weather will be like.
3. Which apps do you use?
   a. Have you ever used an app related to health, nutrition, or sports?
4. Have you ever heard the words "artificial intelligence"? If so, where?
   a. e.g., WhatsApp, ChatGPT, etc.
   b. If necessary, briefly explain the term: Artificial intelligence is like a smart computer that can learn to do things, such as playing games or answering questions, without a human having to tell it what to do every time. It's like a robot that can think and solve problems on its own.

C) Main section – Scenario 1

Since you were born, you have visited your pediatrician several times. Doctors help you when you are sick. But they do more than that: they also check carefully to see if you are developing normally.

This means, for example, that they check how much you weigh, how you move and how you speak – but also whether you feel comfortable at kindergarten or school, whether you have friends, and whether you tend to be happy or sad. If there are any problems, the doctors will try to help you with your development. For example, if you have trouble speaking, they might arrange for a professional to help you practice speaking.

Now imagine there was an app that you and your parents could use to monitor your development. The app could let you know if you are developing differently than normal or if there are signs that you are not living a healthy lifestyle.

a. Do you have any questions regarding comprehension? Is anything in the description unclear to you? (If there are questions, e.g., regarding specific details of the content, refer to the further focus group discussion; do not address them here.)

Data collection

1. What data, i.e., what information from your cell phone, could the app use?
    a. e.g., things you write directly into the app (manual data entry), videos, audio recordings, things you write on WhatsApp, what you search for on Google, etc.

Benefit

2. How do you think such an app could help you?
3. Would you like to know if something in your development is not normal?

Supervision of the child

4. How much of what the app says should your parents see?
    a. What information about you should your parents definitely not receive?
5. Would you like the app to ask you when information is sent to your parents?
6. Would you like your pediatrician to see what the app says?
    a. What information about you should your doctor not receive under any circumstances?
7. Would you like the app to ask you beforehand when certain information is sent to your doctor?

Fears/apprehensions

8. What would have to happen for you to stop using the app or even delete it?
    a. For example, if it sends too many messages? Or is too complicated?
9. Would you be afraid to be the only one in your circle of friends using this app?

C) Main section – Scenario 2

Now I would like to talk to you about another app. Imagine you have been diagnosed with an illness that will not go away, such as diabetes or epilepsy. Now there is an app that can help you manage your illness. For example, it could tell you when to take your medication. Or it could alert you if there are signs of a problem and you need to see your doctor.

a. Do you have any questions about understanding? Is anything in the description unclear? (If there are questions, e.g., about specific details of the content, refer to the further focus group discussion; do not address them here.) If necessary, give further examples of chronic illnesses.

Data collection

1. What data, i.e., what information from your cell phone, could the app use?
    a. For example, information that you enter directly into the app (manual data entry), videos, audio recordings, information that you write on WhatsApp, what you search for on Google, etc.

Benefit

2. How do you think such an app could help you?
3. Would you want the app to tell you if there is anything you could do to improve the treatment of your illness?
    a. Why/Why not?
4. Would you use the app if it meant you had to go to the doctor less often?

Supervision of the child

5. Who should know what the app says?
    a. You, your parents or your doctor?
6. Would you like the app to ask you when information is sent to your parents or your doctor?

Fears/apprehensions

7. What would have to happen for you to stop using the app or even delete it?
8. Would you be afraid to be the only one in your circle of friends using this app?

C) Main section – General information

Now I have a few more questions.

Access options

1. Could you imagine using the app on your own phone or rather on your parents' phone?
2. Would you feel more comfortable talking to an app than to a doctor?

Function

Imagine you are the inventor of an app that deals with your development or health.

3. What would be important to you in your app?
    a. E.g., attractive appearance, ease of use
4. What should the app be able to do?
    a. Example: explain things, talk to a doctor, be able to speak (chatbot)

Responsibility

5. Would you want a doctor to review the results of an app?
6. If the app gives a result or diagnoses a disease but your doctor disagrees, who would you trust more?

Dealing with misdiagnoses

7. How certain should the app be before it informs you that, for example, something is unusual or that you may have an illness?
    a. If applicable, in percent

Right not to know

8. Should the app tell you everything it evaluates, or would you prefer to decide what it shares with you?
9. Are there things that only your doctor should know so that they can discuss them with your parents in person? Or vice versa?

Transparency

10. Would you like to understand how such an app works, or is it enough for you to see the evaluation?
    a. Explanation, if necessary: Artificial intelligence uses various techniques to determine whether something is likely or unlikely. This is similar to math, where there are sometimes different ways of calculating to arrive at a solution. Would you like to understand the calculation method, or is it enough for you to know the solution?

Data protection

11. When you use such apps, a lot of information about you is stored. Would you agree to the data being anonymized (i.e., without your name) and used, for example, for research projects or the further development of the app?

D) Outlook/Exit

12. Have we forgotten anything? Would you like to add anything that we haven't discussed yet?

Next steps: The groups' responses will be evaluated to create questionnaires so that a large number of people can be asked how they feel about the use of artificial intelligence in such apps.

Thank you very much for participating!
