# Supplementary material for: Children and adolescents‘ views on artificial intelligence in pediatric healthcare: a qualitative focus group study
Source: BMC Pediatr. 2026 Jun 13;26:563. doi: 10.1186/s12887-026-07121-w (PMC13267202; doi:10.1186/s12887-026-07121-w)
Supplement: Supplementary file 2 — Supplementary Material 2. [file 12887_2026_7121_MOESM2_ESM.pdf]

BMC Pediatrics

Children and adolescents‘ views on artificial intelligence in pediatric healthcare: a qualitative focus group study

Lisa Reinhart, MD[1]; Janna-Lina Kerth, MD[1]; Anne C. Bischops, MD[1,2]; Maurus Hagemeister, MD[1]; Lisa Krassuski, BA, MD[1]; Ertan Mayatepek, MD[1]; Thomas Meissner, MD[1]

Affiliations:

1 Department of General Pediatrics, Neonatology and Pediatric Cardiology, Medical Faculty, University Hospital Duesseldorf, Heinrich-Heine-University, Duesseldorf, Germany

2 Computational Health Informatics Program, Boston Children’s Hospital, Boston, MA, USA

Address Correspondence to:

Lisa Reinhart, Department of General Pediatrics, Neonatology and Pediatric Cardiology, Medical Faculty and University Children’s Hospital Duesseldorf

Moorenstr. 5, 40227 Duesseldorf, Germany

Email address: lisa.reinhart@med.uni-duesseldorf.de

Phone: +49 211 81-00

Supplementary Material: Semi-structured interview guideline with adolescents

Focus group discussion

„Acceptance of AI applications in healthcare – adolescents“

A) Introduction

Thank you very much for coming today. My name is XXY, I work at the University Children’s Hospital Duesseldorf, and I will be asking a few questions today. This is XXY. She/he will be taking notes during the conversation.

We are working on a project involving several universities and research centers. Among other things, we want to investigate how new technologies can help pediatricians provide better care for children and adolescents. These new technologies primarily involve what is known as artificial intelligence.

We want to know what different people think about artificial intelligence. For example, what they like about it and what they dislike about it. To find out, we talk to pediatricians, pediatric nurses, parents, children, and teenagers.

Great to have you here today!

Now I’ll explain a few things about how the discussion will proceed.

The discussion will last about one to two hours.

Participation in the discussion is voluntary. You can stop the conversation and leave the room at any time.

The discussion is not a test. There are no right or wrong answers. Everyone should feel free to express their opinions and thoughts during the conversation. It is perfectly okay if you disagree with a group member's opinion.

It is important that only one person speaks at a time.

I have prepared some questions for you. If you think of other important things, you can say them at any time.

Everything that is said during the discussion will be treated confidentially by our team. This means that no one except the people in our team will find out what you say. We ask that you also do not tell anyone else what was said in the discussion.

At the beginning of the discussion, everyone should introduce themselves by their first name. We will anonymize all data. This means that no one will be able to find out who participated in the discussion.

XXY will take notes during the discussion. We will also record the conversation with an audio device. Are you all okay with that? I will turn on the device now.

Do you have any questions before we start the discussion?

Two more notes: Please turn off your cell phones during the discussion or set them to silent. And please help yourselves if you would like something to drink or cookies.

B) Opening

Let's start by briefly introducing ourselves.

My name is XXY (discussion leader), I am XXX years old and I work as XXX. My name is XXY (co-moderator), I am XXX years old and I work as XXX.

1. What are your first names? How old are you?
2. Have you ever used an app related to health?
    a. What was your experience with it?

C) Main section – Scenario 1

Imagine there was an app that you and your parents could use to monitor your development—as a child and now as a teenager. The app uses artificial intelligence to analyze your data. It could alert you if you are not developing as you should, or if there are signs that you might have an illness or be living an unhealthy lifestyle.

a. Do you have any questions regarding comprehension? Is anything in the description unclear to you? (If there are questions, e.g., regarding specific details of the content, refer to the further focus group discussion; do not address them here.)

Data collection

1. What data do you think the app could use?
    a. *Regarding individual suggestions for data sources: What do you think?*
    b. Name any other data sources: manual data entry, videos, audio recordings, sensor data, step counter, and other motion sensors, social media

Benefit

2. How do you think such an app could help you?
3. Would you be more open with the app than with your pediatrician?

Supervision of the child

4. Would you like to decide for yourself whether to use the app, or should your parents decide?
5. What would you think if the app accessed data that you generate when using your cell phone?
    a. For example, what videos you watch on TikTok, what you post on Instagram, what you search for on the internet, what you buy, or what games you play and for how long?
6. How much access should your parents have to the data collected by the app?
    b. What information about you should your parents not be allowed to access under any circumstances?
    c. Would you like the app to ask for your consent before certain data is shared with your parents?
    d. Imagine that the app collects data about how much you exercise or how long you sit at the computer and play games. Should your parents know this?
7. How much access to the data collected by the app should your doctor have?
    a. What information about you should your doctor not receive under any circumstances?
    b. Would you like the app to ask for your consent when certain data is transmitted to your doctor?

Fears/apprehensions

8. Are there any fears or concerns you have about using such an app?
    a. What would have to happen for you to stop using the app or delete it?
    b. Would you use the app if you were the only one in your circle of friends who did?

C) Main section – Scenario 2

Now I would like to talk to you about another app. Imagine you have been diagnosed with a disease, such as diabetes or asthma. Now there is an app that supports you, for example, by monitoring your medication intake or certain values.

a. Do you have any questions about understanding? Is anything in the description unclear to you? (If there are questions, e.g., about specific details of the content, refer to the further focus group discussion; do not address them here.) If necessary, give further examples of chronic illnesses.

Data collection

1. What data do you think the app could use?
    a. Regarding individual suggestions for data sources: What do you think?
    b. Name any other data sources: manual data entry, videos, audio recordings, sensor data, pedometer data, and other motion sensors, social media

Benefit

2. How do you think such an app could help you?
3. Would you be more open with the app than with your pediatrician?
4. Imagine that the app could monitor your illness and treatment and you would have fewer appointments with your family doctor. Would that change your willingness to use the app?

Supervision of the child

In the first scenario, we already asked about the type of access, i.e., what data the app collects and who should have access to this data. This scenario is not about general preventive care, but about managing a chronic illness.

5. Do you think this changes what data the app is allowed to collect?
    a. How would you feel if the app accessed data you generate when using your phone?

    b. For example, what videos you watch on TikTok, what you post on Instagram, what you search for on the internet, what you buy, or what games you play and for how long?

6. Do you think this changes who should have access to data?
    a. Would you like to decide for yourself whether to use the app, or should your parents decide?
    b. How much access should your parents have to the data collected by the app?
    c. What information about you should your parents not be allowed to access under any circumstances?
    d. Would you like the app to ask for your consent before certain data is shared with your parents?
    e. Imagine that the app collects data about how much you exercise or how long you spend playing games on the computer. Should your parents be allowed to know this?
    f. How much access to the data collected by the app should your doctor have?
    g. What information about you should your doctor not be allowed to see under any circumstances?
    h. Would you like the app to ask for your consent before certain data is sent to your doctor?

Fears/apprehensions

7. Are there any fears or concerns you have about using such an app?
    a. What would have to happen for you to stop using the app or delete it?
    b. Would you use the app if you were the only one in your circle of friends who did?

C) Main section – General information

Now I would like to move on to further questions. If your answer refers to only one of the apps presented, please say so.

Access options

1. Would you want to use the app on your own phone or on your parents' phone?

Function

2. Are there any specific features you would like to see in the app?
    a. Example: Explanation of values/abnormalities, networking with doctors, chat function via AI language model (chatbot)

Transparency

3. Artificial intelligence works with probabilities and uses various machine learning methods, such as neural networks. Have you heard of these terms before?
4. What information would you like to receive about how the app processes your data in order to trust it?
    a. Is it enough for you to see the end result, or would you like to know how your data is processed?

Responsibility/Dealing with misdiagnoses

5. Artificial intelligence always works with probabilities. How certain do you think the app should be before it indicates a possible illness or abnormality?
    a. Example: With a probability of 90/50/10%, you have diabetes?
    b. Should the app display probabilities, e.g., percentages, categories such as very certain/certain/rather unlikely...?
6. Should a doctor check the data if the app has found something?
7. Imagine that the app has found an indication that you might have a disease. Who should tell you?
    a. Is it important to you to be able to talk to your doctor about it directly?
8. Who would you trust more if the AI and your doctor disagree about whether there is an abnormality or whether you have a disease?
    a. Why?

Right not to know

9. When your data is analyzed by AI, a lot of information can be obtained. Some of this information may relate to diseases that will only become important later on, or perhaps never. Would you want to know about this?

Influence on medical care

10. Would you feel that the doctor is less competent if he/she is supported in his/her work by an app?
11. Would you feel that the doctor would pay less attention to you if he/she is supported in his/her work by an app?

Data protection

12. When you use such apps, a lot of information about you is stored. Would you agree to this data being anonymized and used, for example, for research projects or to further develop the app?

D) Outlook/Exit

13. Is there anything else we forgot to ask that you would like to share with us?

Next steps: The groups' responses will be evaluated to create questionnaires so that a large number of people can be asked how they feel about the use of artificial intelligence in such apps.

14. Is there anything else you would like to know about this?

Thank you very much for participating!
