# Supplementary material for: Children and adolescents‘ views on artificial intelligence in pediatric healthcare: a qualitative focus group study
Source: BMC Pediatr. 2026 Jun 13;26:563. doi: 10.1186/s12887-026-07121-w (PMC13267202; doi:10.1186/s12887-026-07121-w)
Supplement: Supplementary file 3 — Supplementary Material 3. [file 12887_2026_7121_MOESM3_ESM.pdf]

BMC Pediatrics

Children and adolescents‘ views on artificial intelligence in pediatric healthcare: a qualitative focus group study

Lisa Reinhart, MD[1]; Janna-Lina Kerth, MD[1]; Anne C. Bischops, MD[1,2]; Maurus Hagemeister, MD[1]; Lisa Krassuski, BA, MD[1]; Ertan Mayatepek, MD[1]; Thomas Meissner, MD[1]

Affiliations:

1 Department of General Pediatrics, Neonatology and Pediatric Cardiology, Medical Faculty, University Hospital Duesseldorf, Heinrich-Heine-University, Duesseldorf, Germany

2 Computational Health Informatics Program, Boston Children’s Hospital, Boston, MA, USA

Address Correspondence to:

Lisa Reinhart, Department of General Pediatrics, Neonatology and Pediatric Cardiology, Medical Faculty and University Children’s Hospital Duesseldorf

Moorenstr. 5, 40227 Duesseldorf, Germany

Email address: lisa.reinhart@med.uni-duesseldorf.de

Phone: +49 211 81-00

Supplementary Material: Semi-structured interview guideline with children

Fokusgruppen-Diskussion

„Akzeptanz von KI-Anwendungen im Gesundheitsbereich – Kinder“

A) Einleitung

Vielen Dank, dass ihr heute gekommen seid. Ich bin XXY, arbeite an der Kinderklinik der Uniklinik Düsseldorf und werde heute einige Fragen stellen. Das ist XXY. Sie/Er wird während des Gesprächs Notizen machen.

Wir arbeiten an einem Projekt, bei dem unter anderem erforscht wird, wie neue Techniken dabei helfen können, dass sich Kinderärztinnen und -ärzte besser um Kinder und Jugendliche kümmern können. Bei den neuen Techniken geht es vor allem um die sogenannte künstliche Intelligenz.

Wir wollen gerne wissen, was verschiedene Leute über künstliche Intelligenz denken. Zum Beispiel was sie daran gut und was sie daran schlecht finden. Um das herauszufinden, sprechen wir mit Kinderärztinnen und -ärzten, mit Kinderkrankenschwestern und -pflegern, mit Eltern und mit Kindern und Jugendlichen.

Toll, dass ihr heute mit dabei seid!

Nun erkläre ich noch ein paar Dinge zum Ablauf der Diskussion gleich.

Die Diskussion wird circa eine halbe Stunde bis eine Stunde dauern.

Die Teilnahme an der Diskussion ist freiwillig. Ihr könnt das Gespräch zu jeder Zeit abbrechen.

Die Diskussion ist keine Prüfung oder Test. Es gibt keine falschen und richtigen Antworten. Sagt einfach ganz frei, was ihr denkt und fühlt!

Es ist wichtig, dass immer nur eine Person zur gleichen Zeit spricht.

Ich habe einige Fragen an euch vorbereitet. Wenn euch noch andere wichtige Dinge einfallen, könnt ihr sie zu jeder Zeit sagen.

Alles, was während der Diskussion gesagt wird, wird von unserem Team vertraulich behandelt. Das bedeutet, dass niemand, außer der Leute in unserem Team, erfahren wird, was ihr erzählt. Wir bitten euch darum, auch niemandem davon zu erzählen, was in der Diskussion gesagt wurde.

Zu Beginn des Gesprächs soll sich gleich jeder mit Vornamen vorstellen. Wir werden aber später alle Daten anonymisieren. Das bedeutet, dass niemand herausfinden kann, wer bei der Diskussion mitgemacht hat.

XXY wird während der Diskussion Notizen machen. Außerdem nehmen wir das Gespräch mit einem Audiogerät auf. Seid ihr damit alle einverstanden? Ich schalte das Gerät jetzt ein.

Habt ihr Fragen, bevor wir mit der Diskussion beginnen?

Noch zwei Anmerkungen: Bitte schaltet eure Handys während der Diskussion aus oder stellt sie auf lautlos. Und bitte bedient euch, wenn ihr etwas zu Trinken oder Kekse möchtet.

B) Eröffnung

Fangen wir damit an, uns kurz vorzustellen.

Mein Name ist XXY (Diskussionsleiterin), ich bin XXX Jahre alt und arbeite XXX. Mein Name ist XXY (Ko-Moderatorin), ich bin XXX Jahre alt und arbeite XXX.

1. Wie heißt ihr mit Vornamen? Wie alt seid ihr?
2. Habt ihr ein Handy? Wisst ihr, was eine App ist?
    a. Ggf. kurze Begriffserklärung: *Eine App ist ein Computerprogramm, das man vor allem auf dem Handy oder Tablet nutzt. Es gibt viele verschiedene Apps, mit denen man ganz unterschiedliche Sachen machen kann. Es gibt zum Beispiel Apps zum Spielen, Musik-Apps, Apps zum Chatten, Apps, die sagen, wie das Wetter wird.*
3. Welche Apps benutzt ihr?
    a. Habt ihr schonmal eine App benutzt, in der es um Gesundheit, Ernährung oder Sport ging?
4. Habt ihr schon einmal die Wörter „Künstliche Intelligenz“ gehört? Wenn ja, wo?
    a. z.B. bei WhatsApp, ChatGPT etc.
    b. Ggf. kurze Begriffserklärung: *Künstliche Intelligenz ist wie ein schlauer Computer, der lernen kann, Dinge zu tun, wie z.B. Spiele zu spielen oder Fragen zu beantworten, ohne dass ein Mensch ihm jedes Mal sagen muss, was zu tun ist. Es ist wie ein Roboter, der selbstständig denken und Probleme lösen kann.*

C) Hauptteil – Szenario 1

Seit deiner Geburt warst du schon einige Male bei deinem Kinderarzt oder bei deiner Kinderärztin. Die Ärztinnen und Ärzte helfen dir, wenn du krank bist. Sie machen aber auch noch mehr: sie schauen ganz genau hin, ob du dich normal entwickelst.

Das bedeutet, dass sie zum Beispiel überprüfen, wie viel du wiegst, wie du dich bewegst und wie du sprichst – aber auch, ob du dich im Kindergarten oder der Schule wohl fühlst, ob du Freunde hast oder ob du eher fröhlich oder traurig bist. Wenn es irgendwo Probleme gibt, versuchen die Ärztinnen und Ärzte dir bei deiner Entwicklung zu helfen. Wenn du zum Beispiel Probleme beim Sprechen hast, könnten sie dafür sorgen, dass ein Profi mit dir das Sprechen übt.

Stell dir jetzt vor, es gäbe eine App, mit der du und deine Eltern deine Entwicklung beobachten könnten. Die App könnte euch Bescheid geben, wenn du dich anders als normal entwickelst oder es Hinweise darauf gibt, dass du ungesund lebst.

a. Habt ihr Verständnisfragen? Ist euch etwas aus der Beschreibung unklar? *(Bei Fragen z.B. nach inhaltlichen Konkretisierungen auf die weitere Fokusgruppendiskussion hinweisen, hier noch nicht thematisieren.)*

Datenerhebung

1. Was könntet ihr euch vorstellen, welche Daten, das heißt zum Beispiel welche Sachen von deinem Handy, die App benutzen könnte?
    a. z.B. Sachen, die ihr der App direkt schreibt (manuelle Dateneingab), Videos, Tonaufnahmen, Sachen, die ihr auf WhatsApp schreibt, was ihr bei Google sucht etc.

Nutzen

2. Was glaubt ihr, wie euch eine solche App helfen könnte?
3. Möchtet ihr wissen, wenn etwas in eurer Entwicklung nicht normal ist?

Überwachung des Kindes

4. Wie viel sollen deine Eltern davon sehen, was die App sagt?
    a. Welche Informationen über euch sollen eure Eltern auf gar keinen Fall über euch bekommen?
5. Möchtest du, dass die App dich fragt, wenn Sachen an deine Eltern übermittelt werden?
6. Möchtest du, dass deine Kinderärztin/dein Kinderarzt sieht, was die App sagt?
    a. Welche Informationen über dich soll deine Ärztin/dein Arzt auf gar keinen Fall über dich bekommen?
7. Möchtest du, dass die App dich vorher fragt, wenn bestimmte Informationen an deine Ärztin/deinen Arzt übermittelt werden?

Ängste/Befürchtungen

8. Was müsste passieren, damit du die App nicht nutzen oder sie sogar wieder löschen würdest?
    a. Wenn sie z.B. zu viele Nachrichten schickt? Oder zu aufwendig ist?
9. Hättet ihr Angst, diese App als einzige oder einziger im Freundeskreis zu benutzen?

C) Hauptteil – Szenario 2

Nun möchte ich mit euch über eine andere App sprechen. Stellt euch vor, bei euch wurde eine Krankheit festgestellt, die nicht mehr weggeht, zum Beispiel Diabetes oder Epilepsie. Jetzt gibt es eine App, die euch dabei hilft, mit der Krankheit gut umzugehen. Sie könnte euch zum Beispiel sagen, wann ihr ein Medikament nehmen sollt. Oder auch, wenn es Hinweise für ein Problem gibt, und ihr zur Ärztin oder zum Arzt gehen solltet.

a. Habt ihr Verständnisfragen? Ist euch etwas aus der Beschreibung unklar? *(Bei Fragen z.B. nach inhaltlichen Konkretisierungen auf die weitere Fokusgruppendiskussion*

hinweisen, hier noch nicht thematisieren.), ggf weitere Beispiele für chronische Erkrankungen nennen.

Datenerhebung

1. Was könntet ihr euch vorstellen, welche Daten, das heißt zum Beispiel welche Sachen von deinem Handy, die App benutzen könnte?
    a. z.B. Sachen, die ihr der App direkt schreibt (manuelle Dateneingab), Videos, Tonaufnahmen, Sachen, die ihr auf WhatsApp schreibt, was ihr bei Google sucht etc.

Nutzen

2. Was glaubt ihr, wie euch eine solche App helfen könnte?
3. Würdest du wollen, dass die App dir sagt, wenn du bei der Behandlung der Krankheit etwas verbessern könntest?
    a. Warum/warum nicht?
4. Würdest du die App nutzen, wenn du dadurch weniger zur Ärztin oder zum Arzt gehen müsstest?

Überwachung des Kindes

5. Wer soll erfahren, was die App sagt?
    a. Du, deine Eltern, deine Ärztin/dein Arzt?
6. Möchtest du, dass die App dich fragt, wenn Sachen an deine Eltern oder deine Ärztin/deinen Arzt übermittelt werden?

Ängste/Befürchtungen

7. Was müsste passieren, damit du die App nicht nutzen oder sie sogar wieder löschen würdest?
8. Hättet ihr Angst, diese App als einzige oder einziger im Freundeskreis zu benutzen?

C) Hauptteil – Allgemeines

Nun habe ich noch ein paar weitere Fragen.

Zugangsmöglichkeiten

9. Könntest du dir vorstellen, die App auf deinem eigenen Handy zu benutzen oder eher auf dem Handy deiner Eltern?
10. Würdest du dich eher trauen, mit einer App zu sprechen als mit einer Ärztin/einem Arzt?

Funktion

Stell dir vor, du bist Erfinder oder Erfinderin von einer App, die sich mit deiner Entwicklung oder Gesundheit beschäftigt.

11. Was wäre dir bei deiner App wichtig?
    a. Z.B. schönes Aussehen, leichte Bedienbarkeit
12. Was soll die App können?
    a. Beispiel: Sachen erklären, mit Ärztin/Arzt sprechen, sprechen können (Chatbot)

Verantwortung

13. Würdest du wollen, dass die Auswertung einer App von einer Ärztin/einem Arzt angeschaut wird?

14. Wenn die App ein Ergebnis oder eine Krankheit sagt aber deine Ärztin/dein Arzt anderer Meinung ist, wem würdest du eher vertrauen?

Umgang mit Fehldiagnosen

15. Wie sicher soll sich die App sein, bevor sie dir Bescheid sagt, dass z.B. etwas auffällig ist oder du vielleicht eine Krankheit hast?
    a. Ggf. in Prozent

Recht auf Nichtwissen

16. Soll die App dir alles sagen, was sie auswertet, oder möchtest du entscheiden können, was sie dir mitteilt?
17. Gibt es Dinge, die nur deine Ärztin/dein Arzt erfahren soll, um dann persönlich mit deinen Eltern darüber zu sprechen? Oder andersherum?

Transparenz

18. Möchtest du verstehen, wie so eine App funktioniert oder reicht es dir, die Auswertung zu sehen?
    a. Ggf Erklärung: *Künstliche Intelligenz benutzt verschiedene Techniken, um herauszufinden, ob etwas wahrscheinlich oder nicht so wahrscheinlich ist. Das ist ähnlich wie in Mathe, wo es manchmal verschiedene Rechenwege gibt, um auf eine Lösung zu kommen. Möchtest du den Rechenweg verstehen oder reicht es dir, wenn du die Lösung kennst?*

Datenschutz

19. Wenn du solche Apps nutzt, werden viele Informationen über dich gespeichert. Würdest du zustimmen, dass die Daten anonymisiert (also ohne deinen Namen) zum Beispiel für Forschungsprojekte oder die Weiterentwicklung der App genutzt werden?

D) Ausblick/Ausstieg

20. Haben wir noch etwas vergessen? Möchtet ihr etwas ergänzen, was wir noch nicht besprochen haben?

Weiterer Ablauf: Antworten der Gruppen werden ausgewertet, um Fragebögen zu erstellen, damit ganz viele Menschen dazu befragt werden, wie sie den Einsatz von künstlicher Intelligenz bei solchen Apps finden.

Vielen Dank für eure Teilnahme!
