# Supplementary material for: Children and adolescents‘ views on artificial intelligence in pediatric healthcare: a qualitative focus group study
Source: BMC Pediatr. 2026 Jun 13;26:563. doi: 10.1186/s12887-026-07121-w (PMC13267202; doi:10.1186/s12887-026-07121-w)
Supplement: Supplementary file 4 — Supplementary Material 4. [file 12887_2026_7121_MOESM4_ESM.pdf]

BMC Pediatrics

Children and adolescents‘ views on artificial intelligence in pediatric healthcare: a qualitative focus group study

Lisa Reinhart, MD[1]; Janna-Lina Kerth, MD[1]; Anne C. Bischops, MD[1,2]; Maurus Hagemeister, MD[1]; Lisa Krassuski, BA, MD[1]; Ertan Mayatepek, MD[1]; Thomas Meissner, MD[1]

Affiliations:

1 Department of General Pediatrics, Neonatology and Pediatric Cardiology, Medical Faculty, University Hospital Duesseldorf, Heinrich-Heine-University, Duesseldorf, Germany

2 Computational Health Informatics Program, Boston Children’s Hospital, Boston, MA, USA

Address Correspondence to:

Lisa Reinhart, Department of General Pediatrics, Neonatology and Pediatric Cardiology, Medical Faculty and University Children’s Hospital Duesseldorf

Moorenstr. 5, 40227 Duesseldorf, Germany

Email address: lisa.reinhart@med.uni-duesseldorf.de

Phone: +49 211 81-00

Supplementary Material: Semi-structured interview guideline with adolescents

Fokusgruppen-Diskussion

„Akzeptanz von KI-Anwendungen im Gesundheitsbereich – Jugendliche“

A) Einleitung

Vielen Dank, dass ihr heute gekommen seid. Ich bin XXY, arbeite an der Kinderklinik der Uniklinik Düsseldorf und werde heute einige Fragen stellen, um die Diskussion anzuregen. Das ist XXY. Sie/Er wird während des Gesprächs Notizen machen.

Wir arbeiten an einem Projekt, bei dem mehrere Universitäten und Forschungszentren mitmachen. In dem Projekt wollen wir unter anderem erforschen, wie neue Techniken dabei helfen können, dass sich Kinderärztinnen und -ärzte besser um Kinder und Jugendliche kümmern können. Bei den neuen Techniken geht es vor allem um die sogenannte künstliche Intelligenz.

Wir wollen gerne wissen, was verschiedene Leute über künstliche Intelligenz denken. Zum Beispiel was sie daran gut und was sie daran schlecht finden. Um das herauszufinden, sprechen wir mit Kinderärztinnen und -ärzten, mit Kinderkrankenschwestern und -pflegern, mit Eltern und mit Kindern und Jugendlichen.

Toll, dass ihr heute mit dabei seid!

Nun erkläre ich noch ein paar Dinge zum Ablauf der Diskussion gleich.

Die Diskussion wird circa ein bis zwei Stunden dauern.

Die Teilnahme an der Diskussion ist freiwillig. Ihr könnt zu jeder Zeit das Gespräch abbrechen und den Raum verlassen.

Die Diskussion ist keine Prüfung. Es gibt keine falschen und richtigen Antworten. Jeder soll während des Gesprächs seine Meinungen und Gedanken frei äußern. Es ist vollkommen in Ordnung, wenn ihr mit der Meinung eines Gruppenmitglieds nicht übereinstimmt.

Es ist wichtig, dass immer nur eine Person zur gleichen Zeit spricht.

Ich habe einige Fragen an euch vorbereitet. Wenn euch noch andere wichtige Dinge einfallen, könnt ihr sie zu jeder Zeit sagen.

Alles, was während der Diskussion gesagt wird, wird von unserem Team vertraulich behandelt. Das bedeutet, dass niemand, außer der Leute in unserem Team, erfahren wird, was ihr erzählt. Wir bitten euch darum, auch niemandem davon zu erzählen, was in der Diskussion gesagt wurde.

Zu Beginn des Gesprächs soll sich gleich jeder mit Vornamen vorstellen. Wir werden alle Daten anonymisieren. Das bedeutet, dass niemand herausfinden kann, wer bei der Diskussion mitgemacht hat.

XXY wird während der Diskussion Notizen machen. Außerdem nehmen wir das Gespräch mit einem Audiogerät auf. Seid ihr damit alle einverstanden? Ich schalte das Gerät jetzt ein.

Habt ihr Fragen, bevor wir mit der Diskussion beginnen?

Noch zwei Anmerkungen: Bitte schaltet eure Handys während der Diskussion aus oder stellt sie auf lautlos. Und bitte bedient euch, wenn ihr etwas zu Trinken oder Kekse möchtet.

B) Eröffnung

Fangen wir damit an, uns kurz vorzustellen.

Mein Name ist XXY (Diskussionsleiterin), ich bin XXX Jahre alt und arbeite XXX. Mein Name ist XXY (Ko-Moderatorin), ich bin XXX Jahre alt und arbeite XXX.

1. Wie heißt ihr mit Vornamen? Wie alt seid ihr?
2. Habt ihr schon einmal eine App benutzt, bei der es um Gesundheit ging?
    a. Welche Erfahrungen habt ihr damit gemacht?

C) Hauptteil – Szenario 1

Stell dir vor, es gäbe eine App, mit der du und deine Eltern deine Entwicklung – als Kind und jetzt als Jugendliche/Jugendlicher – beobachten könnten. Die App funktioniert mit künstlicher Intelligenz, die deine Daten auswertet. Sie könnte euch Hinweise darauf geben, wenn du dich nicht so entwickelst, wie es normal wäre oder es Hinweise darauf gibt, dass du eine Krankheit haben könntest oder ungesund lebst.

a. Habt ihr Verständnisfragen? Ist euch etwas aus der Beschreibung unklar? *(Bei Fragen z.B. nach inhaltlichen Konkretisierungen auf die weitere Fokusgruppendiskussion hinweisen, hier noch nicht thematisieren.)*

Datenerhebung

1. Was könntet ihr euch vorstellen, welche Daten die App benutzen könnte?
    a. *Zu einzelnen Vorschlägen von Datenquellen:* Wie fändet ihr das?
    b. Ggf weitere Datenquellen nennen: manuelle Dateneingabe, Videos, Tonaufnahmen, Sensordaten, Schrittzählerdaten und andere Bewegungssensoren, Social Media

Nutzen

2. Was glaubt ihr, wie euch eine solche App helfen könnte?
3. Würdet ihr euch der App mehr öffnen als eurer Kinderärztin/eurem Kinderarzt?

Überwachung des Kindes

4. Möchtest du selbst darüber entscheiden, ob du die App benutzt, oder dürfen das deine Eltern entscheiden?
5. Wie fändet ihr es, wenn die App auf Daten, die ihr bei der Nutzung des Handys generiert, zugreifen würde?
    a. Zum Beispiel, welche Videos ihr auf TikTok anschaut, was ihr auf Instagram postet, wonach ihr im Internet sucht, was ihr einkauft oder was und wie lange ihr so spielt?
6. Wie viel Zugriff auf die Daten, die die App erhebt, sollen eure Eltern haben?
    a. Welche Informationen über dich sollen eure Eltern auf gar keinen Fall über euch bekommen?
    b. Möchtet ihr, dass die App nach deiner Zustimmung fragt, wenn bestimmte Daten an deine Eltern übermittelt werden?
    c. Stell dir vor, die App würde Daten darüber erheben, wie viel du dich bewegst oder wie lange du am Computer sitzt und zockst. Dürfen eure Eltern das wissen?
7. Wie viel Zugriff auf die Daten, die die App erhebt, soll eure Ärztin/euer Arzt haben?
    a. Welche Informationen über euch soll eure Ärztin/euer Arzt auf gar keinen Fall über dich bekommen?
    b. Möchtet ihr, dass die App nach eurer Zustimmung fragt, wenn bestimmte Daten an eure Ärztin/euren Arzt übermittelt werden?

Ängste/Befürchtungen

8. Gibt es Ängste oder Befürchtungen, die ihr mit der Nutzung solch einer App verbindet?
    a. Was müsste passieren, damit ihr die App nicht nutzt oder sie wieder löschen würdet?
    b. Würdest du die App benutzen, wenn du die oder der einzige in deinem Freundeskreis wärst?

C) Hauptteil – Szenario 2

Nun möchte ich mit euch über eine andere App sprechen. Stellt euch vor, bei euch wurde eine Krankheit festgestellt, zum Beispiel Diabetes oder Asthma. Nun gäbe es eine App, die euch dabei begleitet, also zum Beispiel die Einnahme von Medikamenten oder bestimmte Werte überwacht.

a. Habt ihr Verständnisfragen? Ist euch etwas aus der Beschreibung unklar? *(Bei Fragen z.B. nach inhaltlichen Konkretisierungen auf die weitere Fokusgruppendiskussion hinweisen, hier noch nicht thematisieren.), ggf weitere Beispiele für chronische Erkrankungen nennen.*

Datenerhebung

1. Was könntet ihr euch vorstellen, welche Daten die App benutzen könnte?
    a. *Zu einzelnen Vorschlägen von Datenquellen:* Wie fändet ihr das?
    b. Ggf weitere Datenquellen nennen: manuelle Dateneingabe, Videos, Tonaufnahmen, Sensordaten, Schrittzählerdaten und andere Bewegungssensoren, Social Media

Nutzen

2. Was glaubt ihr, wie euch eine solche App helfen könnte?
3. Würdet ihr euch der App mehr öffnen als eurer Kinderärztin/Kinderarzt?
4. Stellt euch vor, die App kann eure Krankheit und eure Therapie überwachen und ihr hättet weniger Termine bei eurer Hausärztin/Hausarzt. Würde das etwas an eurer Bereitschaft, die App zu nutzen, ändern?

Überwachung des Kindes

Wir haben im ersten Szenario schon nach der Art und Weise des Zugriffs gefragt, also zum Beispiel welche Daten die App erheben und wer Zugriff auf diese Daten haben sollte. In diesem Szenario geht es nicht um die allgemeine Vorsorge, sondern um die Begleitung einer chronischen Krankheit.

5. Findet ihr, das ändert etwas daran, welche Daten die App erheben darf?
    a. Wie fändet ihr es, wenn die App auf Daten, die ihr bei der Nutzung des Handys generiert, zugreifen würde?
    b. Zum Beispiel, welche Videos ihr auf TikTok anschaut, was ihr auf Instagram postet, wonach ihr im Internet sucht, was ihr einkauft oder was und wie lange ihr so spielt?

6. Findet ihr, das ändert etwas daran, wer Zugriff auf Daten haben sollte?
    a. Möchtest du selbst darüber entscheiden, ob du die App benutzt, oder dürfen das deine Eltern entscheiden?
    b. Wie viel Zugriff auf die Daten, die die App erhebt, sollen eure Eltern haben?
    c. Welche Informationen über dich sollen eure Eltern auf gar keinen Fall über euch bekommen?
    d. Möchtet ihr, dass die App nach deiner Zustimmung fragt, wenn bestimmte Daten an deine Eltern übermittelt werden?
    e. Stell dir vor, die App würde Daten darüber erheben, wie viel du dich bewegst oder wie lange du am Computer sitzt und zockst. Dürfen eure Eltern das wissen?
    f. Wieviel Zugriff auf die Daten, die die App erhebt, soll eure Ärztin/euer Arzt haben?
    g. Welche Informationen über euch soll eure Ärztin/euer Arzt auf gar keinen Fall über dich bekommen?
    h. Möchtet ihr, dass die App nach eurer Zustimmung fragt, wenn bestimmte Daten an eure Ärztin/euren Arzt übermittelt werden?

Ängste/Befürchtungen

7. Gibt es Ängste oder Befürchtungen, die ihr mit der Nutzung solch einer App verbindet?
    a. Was müsste passieren, damit ihr die App nicht nutzt oder sie wieder löschen würdet?
    b. Würdest du die App benutzen, wenn du die oder der einzige in deinem Freundeskreis wärst?

C) Hauptteil – Allgemeines

Nun möchte ich zu weiteren Fragen kommen. Wenn ihr euch bei eurer Antwort nur auf eine der vorgestellten Apps bezieht, dann sagt das bitte.

Zugangsmöglichkeiten

1. Würdest du die App auf deinem eigenen Handy benutzen wollen oder eher auf dem Handy deiner Eltern?

Funktion

2. Gibt es bestimmte Funktionen, die ihr euch bei der App wünschen würdet?
    a. Beispiel: Erklärung von Werten/Auffälligkeiten, Vernetzung mit ÄrztInnen, Chatfunktion über KI-Sprachmodell (Chatbot)

Transparenz

3. Künstliche Intelligenz arbeitet mit Wahrscheinlichkeiten und wendet dabei verschiedene Methoden des maschinellen Lernens an, zum Beispiel neuronale Netzwerke. Habt ihr von diesen Begriffen schon etwas gehört?
4. Welche Informationen möchtet ihr darüber bekommen, wie die App eure Daten verarbeitet, um ihr zu vertrauen?
    a. Reicht es dir zu sehen, was am Ende herauskommt, oder möchtest du wissen, wie deine Daten verarbeitet werden?

Verantwortung/Umgang mit Fehldiagnosen

5. Eine künstliche Intelligenz arbeitet immer mit Wahrscheinlichkeiten. Wie sicher soll sich die App eurer Meinung nach sein, bevor sie auf eine mögliche Krankheit oder Auffälligkeit hinweist?
    a. Beispiel: Mit einer Wahrscheinlichkeit von 90/50/10% leidet ihr an Diabestes?
    b. Sollte die App Wahrscheinlichkeitsangaben aufführen, z.B. Prozente, Kategorien wie sehr sicher/sicher/eher unwahrscheinlich…?
6. Soll eine Ärztin oder ein Arzt die Daten überprüfen, wenn die App etwas herausgefunden hat?
7. Stellt euch vor, die App hat einen Hinweis darauf gefunden, dass du eine Krankheit haben könntest. Wer soll dir das mitteilen?
    a. Ist es dir wichtig, direkt mit deiner Ärztin/deinem Arzt darüber sprechen zu können?
8. Wem würdet ihr eher vertrauen, wenn die KI und eure Ärztin/euer Arzt unterschiedlicher Meinung sind, ob es eine Auffälligkeit gibt oder ihr eine Krankheit habt?
    a. Warum?

Recht auf Nichtwissen

9. Bei der Auswertung von euren Daten durch eine KI kann man viele Informationen erhalten. Einige davon betreffen vielleicht Krankheiten, die erst später oder nie wichtig werden. Würdet ihr darüber Bescheid wissen wollen?

Einfluss auf ärztliche Betreuung

10. Hättet ihr das Gefühl, die Ärztin/der Arzt ist weniger kompetent, wenn er/sie in der Arbeit durch eine App unterstützt wird?
11. Hättet ihr das Gefühl, die Ärztin/der Arzt würde sich euch weniger zuwenden, wenn sie/er in der Arbeit durch eine App unterstützt wird?

Datenschutz

12. Wenn ihr solche Apps nutzt, werden viele Informationen über euch gespeichert. Würdet ihr zustimmen, dass die Daten anonymisiert zum Beispiel für Forschungsprojekte oder die Weiterentwicklung der App genutzt werden?

D) Ausblick/Ausstieg

13. Haben wir noch etwas vergessen zu fragen, was ihr uns gerne mit auf den Weg geben möchtet?

Weiterer Ablauf: Antworten der Gruppen werden ausgewertet, um Fragebögen zu erstellen, damit ganz viele Menschen dazu befragt werden, wie sie den Einsatz von künstlicher Intelligenz bei solchen Apps finden.

14. Möchtet ihr hierzu noch etwas wissen?

Vielen Dank für eure Teilnahme!
